# All-passive nonreciprocal metastructure

**DOI:** 10.1038/ncomms9359

**Published:** 2015-09-28

**Authors:** Ahmed M. Mahmoud, Arthur R. Davoyan, Nader Engheta

**Affiliations:** 1 Department of Electrical and Systems Engineering, University of Pennsylvania, Philadelphia, Pennsylvania 19104, USA

## Abstract

One-way propagation of light, analogous to the directional flow of electrons in the presence of electric potential difference, has been an important goal in the wave–matter interaction. Breaking time-reversal symmetry in photonic flows is faced with challenges different from those for electron flows. In recent years several approaches and methods have been offered towards achieving this goal. Here we investigate another systematic approach to design all-passive relatively high-throughput metastructures that exhibit nonreciprocal properties and achieve wave-flow isolation. Moreover, we build on those findings and propose a paradigm for a quasi-two-dimensional metastructure that mimics the nonreciprocal property of Faraday rotation without using any magnetic or electric biasing. We envision that the proposed approaches may serve as a building block for all-passive time-reversal symmetry breaking with potential applications for future nonreciprocal systems and devices

## Abstract (web summary)

The design of all-passive nonreciprocal metastructures is a challenging task as there are trade-offs between the nonreciprocal transmission ratio and insertion loss. Here, Mahmoud *et al*. propose a concept for all-passive, high-throughput metastructures that exhibit nonreciprocal properties and wave-flow isolation.

## Graphical abstract

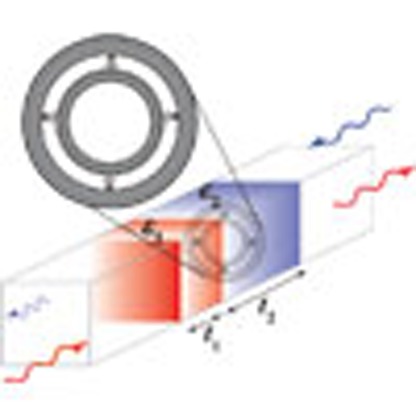

Recent years have witnessed astonishing progress in the design of structures and systems that control and manipulate electromagnetic waves in desired manners^1^,^2^,^3^,^4^,^5^. Of particular interest are ultrathin structures and composites, known as metasurfaces, that exhibit electromagnetic properties not readily attainable in nature^6^,^7^,^8^. The interest towards these systems has sparkled mostly because of a variety of promising applications in various ranges of electromagnetic spectrum: metasurfaces may be useful devices in today's microwave technology, where compact antennas, ultrathin layers with extreme chirality and asymmetric transmission have been proposed and fabricated^9^,^10^,^11^; at optical frequencies, metasurfaces may be the key towards the next generation of nanophotonics with various potential applications^6^,^7^,^12^,^13^,^14^; and even the THz and graphene-based technology is widely exploiting the metasurface principles^15^,^16^,^17^.

Metasurfaces are usually constituted of regular metal and dielectric materials specifically crafted to give a desired electromagnetic response. It is the combination of the shape and dispersion properties of the inclusions that determines the properties of the whole system. Exploiting materials with nontrivial electromagnetic properties may significantly enhance the functionality and introduce new degrees of freedom for unprecedented features. For instance, gyrotropic materials that are sensitive to the magnetic biasing may be employed to tune and control the transmission properties of the system. More specifically, metastructures with gyrotropic properties exhibit time-reversal asymmetry and may be exploited for nonreciprocal propagation and transmission, for example, Faraday rotation of light polarization and asymmetric transmission for opposite directions of illumination—the latter is a property that is crucial for the electromagnetic wave isolation^18^,^19^,^20^,^21^,^22^,^23^. The ability to break the transmission symmetry is, however, not limited to the use of gyortropic materials; it is also possible by utilizing time-dependent properties to break the system's reciprocity both in optical^24^,^25^ and acoustic domains^26^. However, the aforementioned scenarios may lead to relatively bulky device designs. Recently, metasurfaces with active electronic elements, that is, transistors, have been proposed and tested for electrically controllable more efficient nonreciprocal behaviour^27^,^28^,^29^. The complexities and limitations in the design and implementation of active elements and their biasing networks do, however, pose an additional complex step in such designs. Such complexities may need to be reduced to simplify future designs. In this framework, developing all-passive metasurfaces that acquire efficient nonreciprocal behaviour is of importance. Such systems may find broad range of applications from energy harvesting and light trapping in solar cells to nonreciprocal decoupling of transmitters and receivers within telecommunication and radar systems, and applications in image-processing techniques. Nonetheless, the design of all-passive nonreciprocal subwavelength metastructures is still a challenging task. Within that context, nonlinear systems may be the key towards the design of passive, nonreciprocal, self-controlled systems dependent solely on the input power level^30^,^31^. In particular, many previous lines of work have proposed both theoretically and experimentally the concept of an electromagnetic diodes or isolators using nonlinearities^30^,^31^,^32^,^33^,^34^,^35^,^36^,^37^,^38^,^39^,^40^,^41^,^42^. (Here we note that one should differentiate the concepts of ‘electromagnetic diode' and ‘optical isolator'—by an electromagnetic diode we consider a two-port system that transmits wave in one direction and reflects it in the other direction. This is in contrast with regular isolators, in which the backward signal is irretrievably ‘lost', that is, is changed into either a different mode, a different frequency, dissipated or other modalities.) In some of these previous lines of work, it was shown that using a combination of nonlinearities and structural asymmetry it is possible to break the symmetry of wave transmission in forward and backward directions and acquire nonreciprocal behaviour. Those systems have usually been relatively large in size and by no means subwavelength. Moreover, to the best of our knowledge, owing to the design principles of such nonlinear systems, a dramatic trade-off between the nonreciprocal transmission ratio (NTR) and the insertion loss (IL) has been experienced. For instance, significant difference in the transmission between forward- and backward-propagating waves (that is, high NTR) has been at the expense of a very low transmission in the forward direction (that is, high IL)^30^,^31^,^32^,^33^,^34^,^35^,^36^,^37^,^38^. At the other extreme having an acceptably low IL was shown to be possible but at the expense of having low NTR^40^,^41^,^42^.

Here we propose a concept for all-passive, not-externally biased, nonreciprocal metasurfaces that does not suffer from the dramatic trade-off mentioned above. Our technique is rather generic and in principle not limited to a specific range of operating frequency. Moreover, building on this approach, we propose a case where a quasi-two-dimensional (2D) metasurface can be used to mimic the nonreciprocal Faraday rotation.

## Results

### Proposed concept

**Figure 1:**
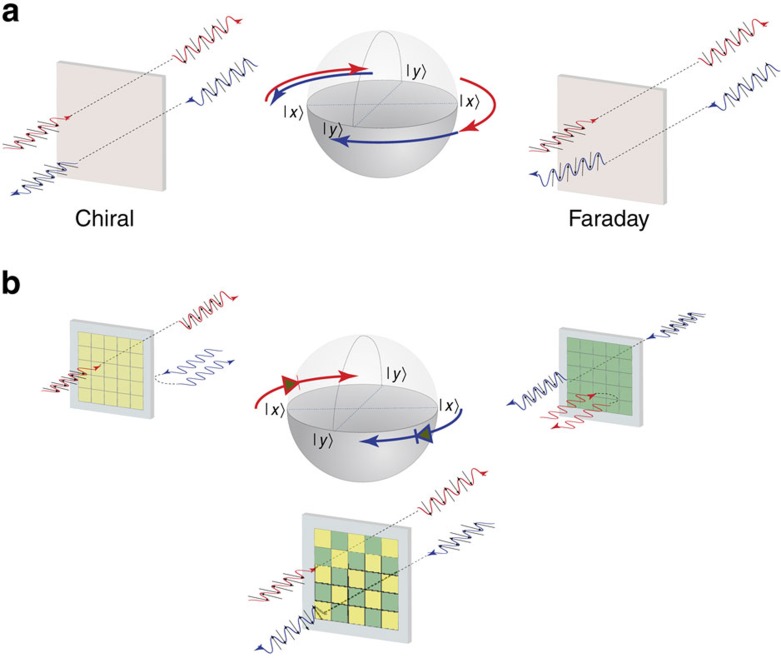
Schematics of all-passive nonreciprocal metastructures. (**a**) The difference between the ‘reciprocal' polarization rotation due to chirality and the ‘nonreciprocal' Faraday rotation phenomenon is shown, both mapped conceptually on the Poincaré sphere. (**b**) Depending on the illumination direction of the incident wave, one of the two constituent designs (shown as green and yellow), acting as a ‘wave diode', allows the wave to go through, interacting with the chiral element (not shown) in the unit. Owing to this interaction, the plane of polarization of the wave rotates clockwise by nearly 90 ° as it goes through this wave diode. The chiral elements in the diodes for waves going into the (+*z*) direction (‘yellow' design) are mirror image of the chiral elements in the diodes for waves going into the (−*z*) direction (‘green' design). The all-passive metastructure formed as the checkerboard pattern of such alternating designs may function as a nonreciprocal metasurface mimicking Faraday rotation, while no biasing electric or magnetic field is used.

Figure 1 shows the proposed concept. The metasurface unit cells consist of an all-passive electromagnetic ‘diode' (transmitting waves in one given direction only, which we will discuss later here) and a chiral element. We design the metastructure in a checkerboard manner considering that two neighbouring unit cells are mirror images of each other. Note that chiral elements in this case lead to the polarization rotation in the opposite directions, see Fig. 1. Consider illuminating the structure from one side. In this case only the unit cells with electromagnetic diodes allowing wave propagation would allow the wave to pass through. The wave in these cells would interact with the chiral structure and acquire a certain degree of polarization rotation (clockwise or anticlockwise depending on the orientation of the chiral element in those units). On the other hand, illuminating the structure from the other side, the other set of unit ‘diode' cells will let the wave go through; however, in these cells the chiral elements are the mirror image of the other chiral element, thus leading to the rotation of the wave polarization in the same direction, which contrasts sharply with conventional chiral metamaterial structures and metasurfaces. Hence, such a system would be mimicking the Faraday rotation of polarization. Clearly, the main challenge here is the design of an all-passive subwavelength electromagnetic diode with high-performance characteristics. This is discussed in the next section.

### All-passive nonlinear electromagnetic ‘diode'

As mentioned earlier, previous designs of an all-passive nonlinear electromagnetic ‘diode' reported in the literature have certain limitations that include the inherently large size of the device in addition to the significant trade-off between the transmission ratio and the IL. Traditionally, optical isolation within such systems is based on the direction-dependent resonance shift in structures with some distributed nonlinearity, for example, a nonlinear defect slab in a photonic crystal for example, ref. 33, or a nonlinear microring resonator combined with the structural asymmetry, for example, ref. 30. The nonlinear layer (or resonator) is mostly of the order of several wavelengths ensuring efficient accumulation of the nonlinear response, for example, nonlinear phase or resonance shift, and is sensitive to the amplitude profile averaged across the whole nonlinear element, rather to the local field amplitude. Hence, in some of those scenarios one needs to design the structural asymmetry in such a way that the average amplitude in the whole nonlinear region is essentially different for forward and backward directions of illuminations. The latter has been typically achieved with the use of evanescent fields that because of their exponential amplitude profile naturally have strong amplitude gradient—placing the nonlinear element in the different parts of the evanescent field it is possible to trigger different nonlinear responses, that is, achieve very high NTR. However, the transmission based on evanescent fields exploiting the tunnelling mechanism is essentially low, leading to high IL. (We like to mention here that somewhat similar asymmetry mechanism is used in gain–loss, that is, PT-symmetric, systems^37^,^38^,^39^). In contrast, here we show that utilizing subwavelength nonlinear elements and exploiting local nonlinear response (that is, response to the local amplitude of the electric field) it is possible to operate with propagating waves, rather than the evanescent waves and thus achieve higher performance. Importantly, our design principle allows the possibility to reduce the size of the system significantly while enhancing the performance of our nonlinear diode by making the above-mentioned trade-off between having a low IL and a high NTR less dramatic. We stress that this approach is rather general and may in principle be exploited in different frequency regimes, including optical frequencies, provided that the subwavelength nonlinear resonant system is utilized^43^,^44^,^45^,^46^.

**Figure 2:**
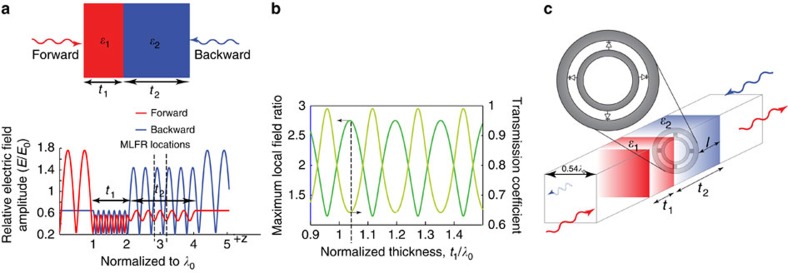
Concept of the proposed electromagnetic wave ‘diode'. (**a**) The relative electric field amplitude distribution within a bilayered asymmetric one-dimensional (1D) slab infinitely extent in the transverse directions (normalized to incident field amplitude *E*_0_). While the paired slab is reciprocal, the amplitude of the total electric field distribution in the partial standing waves within the structure is dependent on the side from which it is excited. The red and blue curves show the field amplitude distribution normalized to incident field amplitude *E*_0_ for the forward (+z)- and backward (−z)-propagating waves, respectively. The dashed lines show two of the locations of MLFR, where the ratio between the local field amplitude for the forward and backward illuminations is maximum. These are the planes where nonlinearly loaded resonator should be inserted for the most efficient nonlinear response. (**b**) MLFR (green curve) and transmission coefficient (yellow curve) versus normalized thickness *t*_1_. The dashed line shows the operating point for which the normalized field distribution is shown in **a**. (**c**) Bilayered asymmetric slab consisting of two layers with dielectric permittivities *ɛ*_1_ and *ɛ*_2_ and thicknesses *t*_1_ and *t*_2_ are inserted in the squared-cross-section metallic waveguide. The inset shows the varactor-loaded thin resonator placed at a distance *l* from one of the slab ends where one of the MLFR locations is. The resonator is made of two concentric rings loaded by four varactors distributed symmetrically along its perimeter. The bilayered slab and the ring resonator are placed within a rectangular waveguide.

We begin our analysis with the study of field distributions in a linear bilayered one-dimensional (1D) asymmetric slab infinitely extent in the transverse directions, schematically shown in Fig. 2a. The slab consists of two linear dielectric layers with dielectric permittivities *ɛ*_1_ and *ɛ*_2_ and thicknesses *t*_1_ and *t*_2_, respectively. Illuminating the slab from either side causes the formation of partial standing-wave patterns in the structure. Owing to geometrical asymmetry of the system, the field profiles inside the slab would be generally different for opposite directions of illumination, see Fig. 2a. Note that in such a linear system because of the structure's reciprocity transmission characteristics for both directions of illumination would be identical. However, the local field amplitudes in the partial standing-wave regions for the forward and backward directions of illumination are essentially different as depicted in Fig. 2a. Furthermore, inside the slab, positions, where the local field amplitudes ratio between the cases of forward- and backward-propagating waves is maximum, namely maximum local field ratio (MLFR) locations, exist. Therefore, nonlinear, amplitude-dependent, resonant structures with thicknesses much smaller than the wavelength, that is, essentially subwavelength, when placed at those locations may respond differently to the two different directions of illumination. In Fig. 2b, we choose *ɛ*_1_ and *ɛ*_2_ to be 10*ɛ*_0_ and 2*ɛ*_0_, respectively, and *t*_2_ to be 2.03*λ*_0_, where *λ*_0_ is the free space wavelength at the operating frequency. We plot the MLFR along with the corresponding transmission coefficient versus the variation of the relative thickness of the first layer in the bilayer slab (by varying *t*_1_/*λ*_0_). Our analysis shows that when *t*_1_=1.03*λ*_0_, transmission coefficient of ∼0.65 and an MLFR of 2.733 can be achieved. This is the case for which the field distribution is plotted in Fig. 2a. We should emphasize that, while in the main text we show the example of slabs with the thicknesses of *t*_1_=1.03*λ*_0_ and *t*_2_=2.03*λ*_0_ in order for the field distributions to be clearly visible to the reader, our design can be made for the subwavelength-thick slabs as shown in the Supplementary material. (See the Supplementary Note 1 and Supplementary Fig. 1 for an example of thinner (subwavelength-thick) 1D slabs with the same set of dielectric constants in which the same MLFR and transmission coefficient are preserved.) Moreover, a more detailed discussion regarding the relationship and trade-offs between the transmission coefficient and the MLFR can be found in Supplementary Note 1.

Although the above discussion was for the 1D slab infinitely extent in the transverse directions, hereafter without loss of generality and for the sake of simplicity and reduction in numerical complexity in the proof of concept we study wave-flow isolation in a rectangular waveguide at microwave frequencies. The corresponding geometry is shown in Fig. 2c where we have a metallic waveguide with a squared-cross-section 0.54*λ*_0_ × 0.54*λ*_0_. Using the same parameters (thicknesses and permittivities) mentioned above for the bilayered slab, now embedded inside the waveguide, we achieve a transmission coefficient of 0.425 and an MLFR of 4.4 (See the Supplementary Note 2 and Supplementary Fig. 2).

The nonlinear subwavelength resonant structure is now placed at one of MLFR locations, that is, *l*=1.65*λ*_0_, where *l* is the distance from the edge of the slab of permittivity *ɛ*_2_ and thickness *t*_2_ as shown in Fig. 2c. The schematic of our nonlinear subwavelength resonant structure is shown in the inset to Fig. 2c. Here we consider two concentric rings with four nonlinear amplitude-dependent capacitive elements (commonly known as varactors)^47^,^48^,^49^ symmetrically placed between the rings, as shown in the inset of Fig. 2c, considering that the nonlinear element is essentially subwavelength ring resonator (we note that our design can be easily extended to an infinite array of resonators; however, here we consider a ring resonator in a waveguide). More details regarding the dimensions and the behaviour of the resonator can be found in Supplementary Notes 3 and 4 and Supplementary Figs 3–7.

**Figure 3:**
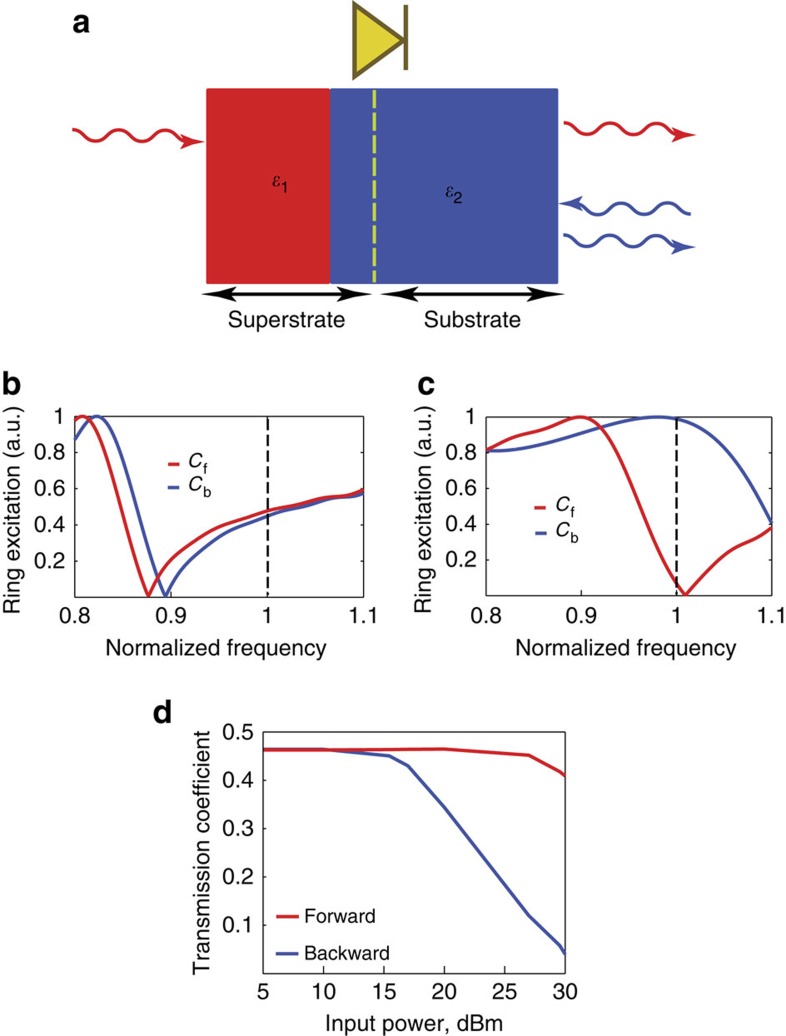
Response of the nonlinearly loaded resonator ring and transmission coefficient of electromagnetic wave diode in the waveguide. (**a**) Schematics of the wave diode. (**b**) The resonance behaviour of the ring (that is, its excited current in arbitrary units (a.u.) versus normalized frequency (with respect to operating frequency), when the ring is located in the waveguide filled with material with *ɛ*_2_=2*ɛ*_0_ for two different values of varactor capacitance. These capacitance values, *C*_f_=0.157 pF and *C*_b_=0.147 pF, are what the varactor experiences when the loaded ring in the bilayered structure and is illuminated with 5 dBm incident power in the forward (+*z*) and backward (−*z*) directions, respectively. In this case, we get almost the same excitation, which yields the same transmission characteristics for both. (**c**) Similar to **b**, except the varactor capacitance values *C*_f_=0.1 pF and *C*_b_=0.05 pF, which are for the case of 30-dBm incident power. Here we note significant difference in the resonance of the ring for different capacitance values, yielding two different transmission coefficients at the operating frequency. (**d**) Transmission coefficient versus input power level. The red and blue curves show the cases of the forward and backward illuminations, respectively.

We choose the dimensions of the nonlinearly loaded resonator such that in its linear state its resonant frequency is detuned from a given operating frequency, implying that the ring is effectively ‘transparent'. For higher intensities we expect a shift of its resonance frequency (because of changing the value of the varactor capacitance) towards the operating frequency with a corresponding decrease in transmission (when the resonance frequency of ring coincides with the operating frequency almost total reflection is expected). Consequently, by inserting such a nonlinear element at the MLFR location within the bilayered dielectric structure in the waveguide, as shown in Figs 2c and 3a, it is possible to tune the transmission properties with respect to the direction of illumination and the power level. It is worth noting that there are, in principle, two resonance phenomena at work here: (1) resonance of the ring and (2) resonance due to the bilayered dielectric slab (that is, Fabry–Perot-type resonance). Figure 3b,c demonstrates how the resonance behaviour of the ring by itself is affected by the different values of capacitance of the varactor, since these capacitance values depend on the power level of the incident wave when the loaded ring is inserted in the bilayered structure. In these two panels, the resonance behaviour of the ring as characterized by its induced current when the ring is embedded in the waveguide filled with only the material with *ɛ*_2_=2*ɛ*_0_, and loaded with different values of capacitance of varactor is shown. The capacitance values, given in the captions, are selected based on the values that the varactor would have when the loaded ring is in the bilayered structure in the waveguide and is illuminated with the incoming wave in the forward and backward directions with different power levels. It is clear that for low power (5 dBm), the varactors are operating in their linear regime having almost the same value of capacitance for propagation in both directions and that there is only a very slight shift between the resonance frequency positions (Fig. 3b), and consequently there is no pronounced difference in the transmission in both directions at the operating frequency. On the other hand, for the power of 30 dBm, the varactor capacitance values are different for different directions of propagation, leading to a major shift in resonance frequencies (Fig. 3c). The final transmission coefficient is influenced by both the resonance of the ring and the resonance of bilayered slab. We solve the nonlinear problem numerically using CST Microwave Studio in a steady-state approximation as discussed in Supplementary Note 3 and Supplementary Fig. 4. Figure 3d shows the calculated transmission coefficients in the structures for forward and backward directions of illumination at different incident power levels. We observe that for low power levels, the transmission properties for both directions of illumination are practically the same, and the structure has the transmission coefficient of ∼0.462 for both cases symmetrically. With the increase in the incident power level we observe that a transmission for backward-propagating wave is decreasing, whereas the transmission in a forward direction is practically not changing. For a 30-dBm incident power we already observe transmission coefficient of ∼0.42 for the forward illumination and a negligible transmission coefficient for the backward case. Moreover, the structure is robust towards changing the field polarization angle. This stability of the structure characteristics towards the change in the incident polarization is due to the symmetry of both the resonator structure, and the distribution of the nonlinear elements around the structure. We note here that, to the best of our knowledge, ref. 50 is the only work where the idea of local nonlinear response is considered for the design of an electromagnetic diode, where an infinitesimally thin layer of nonlinear dielectric is embedded into a composite asymmetric metamaterial photonic crystal. There the isolation is due to an unrealistically high value of the Kerr coefficient (comparable to the linear permittivity of the dielectric), rather than due to the interplay of a resonant subwavelength systems and a local field asymmetry, as we exploit in our work.

We believe that our proposal of an efficient electromagnetic diode can find direct applications within various contexts and frequency regimes. For instance, we envision that our approach may be integrated efficiently within the monolithic microwave integrated circuits platforms. Furthermore, our idea, when extended to the optical domain, may pave the way for efficient on-chip optical logic circuitry and data processing.

## Discussion

**Figure 4:**
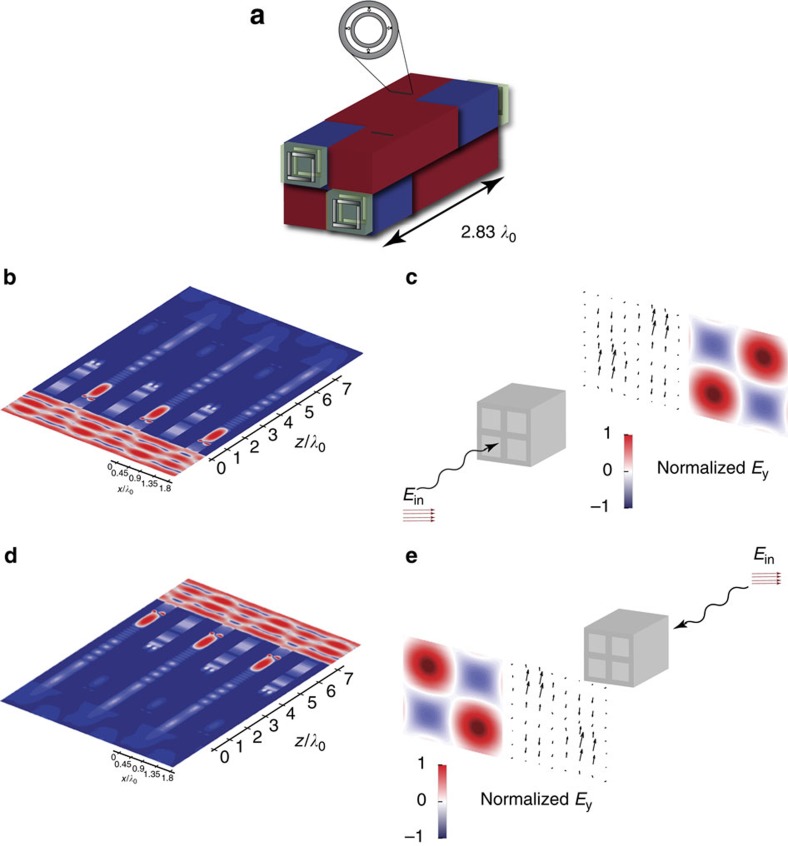
Schematic of all-passive quasi-2D nonreciprocal metastructures. (**a**) Geometry of the device with four units of the checkerboard including two pairs of mirror-imaged chiral elements shown in Fig. 1. (**b**) Cross-section of the electric field intensity distribution within the metastructure with the incident wave propagating in the +*z* direction, (**c**) Simulation results for the output field (shown as compared with the incident field) when the incident wave propagates in the +*z* direction (**d**,**e**) Similar to **b**,**c** but when the incident wave propagates in the −*z* direction.

Having a building block that behaves as an electromagnetic diode exhibiting the intended relatively high-throughput all-passive wave-flow isolation, we can proceed to investigating the intended all-passive quasi-2D metasurface that mimics the nonreciprocal behaviour of Faraday rotation. For the sake of numerical proof of concept, we choose the chiral structure in our unit cells to be the subwavelength bilayered chiral structures from ref. 51, which allows for almost 90° polarization rotation with little reflection. A schematic of the unit cell constituting the metastructure (with two sub-unit cells as discussed before) is shown in Fig. 4a. Figure 4b shows a cross-section of the electric field intensity distribution within the quasi-2D metastructure when illuminated with a plane wave that is linearly polarized along the *x* direction and is propagating in the forward (+*z*) direction. Only the unit cells with the electromagnetic diode oriented to allow wave propagation in the (+*z*) direction are excited. As depicted in Fig. 4c the output's polarization in that case is a rotated version of the incident field's polarization. Illuminating the surface with a plane wave propagating in the opposite (that is, backward (–*z*)) direction instead, only the unit cells with the electromagnetic diodes oriented such that to allow wave propagation in the (–*z*) direction are excited as shown in Fig. 4d. Since the chiral elements in these cells are mirror image of the other chiral elements, we still get a rotated version of the incident wave, but with the same sense of rotation as the one obtained illuminating the surface from the other side as shown in Fig. 4e, mimicking the nonreciprocal Faraday rotation as required while no electric or magnetic bias is used.

In conclusion, we proposed a platform for all-passive metasurfaces with broken time-reversal symmetry. We investigated a systematic design procedure for achieving wave-flow isolation without the usage of any sort of bias (neither magnetic nor electric) that is in principle applicable to any frequency regime. Moreover, we proposed a quasi-2D metastructure that mimics the nonreciprocal Faraday rotation phenomenon exhibiting high optical activity.

Note added: in the final stage of sending our revised manuscript after the review process, we became aware of the June 2015 paper by Shi *et al*.^52^ on limitations of nonlinear optical isolators. The features of our proposed structures reported in the current work are consistent with the findings of Shi *et al*.

## Additional information

**How to cite this article:** Mahmoud, A. M. *et al*. All-passive nonreciprocal metastructure. *Nat. Commun*. 6:8359 doi: 10.1038/ncomms9359 (2015).

## Supplementary Material

Supplementary Information Supplementary Figures 1-7, Supplementary Notes 1-4 and Supplementary References.

## References

[b1] Engheta N. & Ziolkowski R. W. Metamaterials: Physics and Engineering Explorations Wiley (2006).

[b2] Pendry J. B., Schurig D. & Smith D. R. Controlling electromagnetic fields. Science 312, 1780–1782 (2006). 16728597 10.1126/science.1125907

[b3] Soukoulis C. M. & Wegener M. Optical metamaterials — more bulky and less lossy. Science 330, 1633–1634 (2010). 21164003 10.1126/science.1198858

[b4] Pendry J. B. Negative refraction makes a perfect lens. Phys. Rev. Lett. 85, 3966–3969 (2000). 11041972 10.1103/PhysRevLett.85.3966

[b5] Alù A. & Engheta N. Achieving transparency with plasmonic and metamaterial coatings. Phys. Rev. E 72, 016623 (2005). 10.1103/PhysRevE.72.016623 16090123

[b6] Yu N. . Light propagation with phase discontinuities: generalized laws of reflection and refraction. Science 334, 333–337 (2011). 21885733 10.1126/science.1210713

[b7] Ni X., Emani N. K., Kildishev A. V., Boltasseva A. & Shalaev V. M. Broadband light bending with plasmonic nanoantennas. Science 335, 427 (2012). 22194414 10.1126/science.1214686

[b8] Mahmoud A. M., Davoyan A. R. & Engheta N. Nonreciprocal passive metastructure without magnetic bias. IEEE Antennas Propag. Soc. Int. Symp. 496–497 (2013).

[b9] Sievenpiper D., Zhang L., Broas R. F. J., Alexopolous N. G. & Yablonovitch E. High-impedance electromagnetic surfaces with a forbidden frequency band. IEEE Trans. Microw. Theory Tech. 47, 2059–2074 (1999).

[b10] Burokur S. N., Daniel J.-P., Ratajczak P. & de Lustrac A. Tunable bilayered metasurface for frequency reconfigurable directive emissions. Appl. Phys. Lett. 97, 064101 (2010).

[b11] Germain D., Seetharamdoo D., Burokur S. N. & de Lustrac A. Phase-compensated metasurface for a conformal microwave antenna. Appl. Phys. Lett. 103, 124102 (2013).

[b12] Genevet P. . Flat optics: controlling wavefronts with optical antenna metasurfaces. IEEE J. Sel. Top. Quantum Electron. 19, 4700423 (2013).

[b13] Kildishev A. V., Boltasseva A. & Shalaev V. M. Planar photonics with metasurfaces. Science 339, 1232009 (2013). 23493714 10.1126/science.1232009

[b14] Yu N. & Capasso F. Flat optics with designer metasurfaces. Nat. Mater. 13, 139–150 (2014). 24452357 10.1038/nmat3839

[b15] Chen P. Y. . Nanostructured graphene metasurface for tunable terahertz cloaking. New J. Phys. 15, 123029 (2013).

[b16] Fallahi A. & Perruisseau-Carrier J. Design of tunable biperiodic graphene metasurfaces. Phys. Rev. B 86, 195408 (2012).

[b17] Hadad Y., Davoyan A. R., Engheta N. & Steinberg B. Z. Extreme and quantized magneto-optics with graphene meta-atoms and metasurfaces. ACS Photonics 1, 1068–1073 (2014).

[b18] Temnov V. V. . Active magneto-plasmonics in hybrid metal–ferromagnet structures. Nat. Photonics 4, 107–111 (2010).

[b19] Belotelov V. I., Doskolovich L. L. & Zvezdin A. K. Extraordinary magneto-optical effects and transmission through metal-dielectric plasmonic systems. Phys. Rev. Lett. 98, 077401 (2007). 17359058 10.1103/PhysRevLett.98.077401

[b20] Chin J. Y. . Nonreciprocal plasmonics enables giant enhancement of thin-film Faraday rotation. Nat. Commun. 4, 1599 (2013). 23511464 10.1038/ncomms2609

[b21] Fang K., Yu Z., Liu V. & Fan S. Ultracompact nonreciprocal optical isolator based on guided resonance in a magneto-optical photonic crystal slab. Opt. Lett. 36, 4254–4256 (2011). 22048382 10.1364/OL.36.004254

[b22] Yu Z., Wang Z. & Fan S. One-way total reflection with one-dimensional magneto-optical photonic crystals. Appl. Phys. Lett. 90, 121133 (2007).

[b23] Mazor Y. & Steinberg B. Z. Metaweaves: sector-way nonreciprocal metasurfaces. Phys. Rev. Lett. 112, 153901 (2014). 24785040 10.1103/PhysRevLett.112.153901

[b24] Yu Z. & Fan S. Complete optical isolation created by indirect interband photonic transitions. Nat. Photonics 3, 91–94 (2009).

[b25] Manipatruni S., Robinson J. T. & Lipson M. Optical nonreciprocity in optomechanical structures. Phys. Rev. Lett. 102, 213903 (2009). 19519108 10.1103/PhysRevLett.102.213903

[b26] Fleury R., Sounas D. L., Sieck C. F., Haberman M. R. & Alù A. Sound isolation and giant linear nonreciprocity in a compact acoustic circulator. Science 343, 516–519 (2014). 24482477 10.1126/science.1246957

[b27] Kodera T., Sounas D. L. & Caloz C. Artificial Faraday rotation using a ring metamaterial structure without static magnetic field. Appl. Phys. Lett. 99, 031114 (2011).

[b28] Wang Z. . Gyrotropic response in the absence of a bias field. Proc. Natl Acad. Sci. USA 109, 13194–13197 (2012). 22847403 10.1073/pnas.1210923109 PMC3421177

[b29] Sounas D. L., Caloz C. & Alù A. Giant non-reciprocity at the subwavelength scale using angular momentum-biased metamaterials. Nat. Commun. 4, 2407 (2013). 23994940 10.1038/ncomms3407

[b30] Fan L. . An all-silicon passive optical diode. Science 335, 447–450 (2012). 22194410 10.1126/science.1214383 PMC5563475

[b31] Fan L. . Silicon optical diode with 40dB nonreciprocal transmission. Opt. Lett. 38, 1259–1261 (2013). 23595451 10.1364/OL.38.001259

[b32] Fan Y. . Subwavelength electromagnetic diode: one-way response of cascading nonlinear meta-atoms. Appl. Phys. Lett. 98, 151903 (2011).

[b33] Miroshnichenko A. E., Brasselet E. & Kivshar Y. S. Reversible optical nonreciprocity in periodic structures with liquid crystals. Appl. Phys. Lett. 96, 063302 (2010).

[b34] Wang J. . A theoretical model for an optical diode built with nonlinear silicon microrings. J. Light. Technol. 31, 313–321 (2013).

[b35] Shadrivov I. V., Fedotov V. A., Powell D. A., Kivshar Y. S. & Zheludev N. I. Electromagnetic wave analogue of an electronic diode. New J. Phys. 13, 033025 (2011).

[b36] Zhang Y. . Silicon optical diode based on cascaded photonic crystal cavities. Opt. Lett. 39, 1370–1373 (2014). 24690790 10.1364/OL.39.001370

[b37] Chang L. . Parity–time symmetry and variable optical isolation in active–passive-coupled microresonators. Nat. Photonics 8, 524–529 (2014).

[b38] Nazari F., Bender N., Ramezani H. & Kottos T. Optical isolation via PT -symmetric nonlinear Fano resonances. Opt. Express 22, 9574–9584 (2014). 24787845 10.1364/OE.22.009574

[b39] Peng B. . Parity–time-symmetric whispering-gallery microcavities. Nat. Phys. 10, 394–398 (2014).

[b40] Sahoo P. K. & Joseph J. Optical diode using nonlinear polystyrene ring resonators in two-dimensional photonic crystal structure. Appl. Opt. 52, 8252–8257 (2013). 24513826 10.1364/AO.52.008252

[b41] Li N. & Ren J. Non-reciprocal geometric wave diode by engineering asymmetric shapes of nonlinear materials. Sci. Rep. 4, 6228 (2014). 25169668 10.1038/srep06228 PMC4148659

[b42] Kong X., Liu S., Zhang H., Dai Y. & Yang H. A theoretical study of a compact and highly efficient isolator consisting of nonlinear plasma and matching metamaterials. Laser Phys. 23, 055404 (2013).

[b43] Minovich A., Neshev D. N., Powell D. A., Shadrivov I. V. & Kivshar Y. S. Tunable fishnet metamaterials infiltrated by liquid crystals. Appl. Phys. Lett. 96, 193103 (2010).

[b44] Carretero-Palacios S. . Optical switching in metal-slit arrays on nonlinear dielectric substrates. Opt. Lett. 35, 4211–4213 (2010). 21165140 10.1364/OL.35.004211

[b45] Minovich A. . Liquid crystal based nonlinear fishnet metamaterials. Appl. Phys. Lett. 100, 121113 (2012).

[b46] Lee J. . Giant nonlinear response from plasmonic metasurfaces coupled to intersubband transitions. Nature 511, 65–69 (2014). 24990746 10.1038/nature13455

[b47] Boyd R. W. Nonlinear Optics Academic Express (1992).

[b48] Shadrivov I. V., Kozyrev A. B., van der Weide D. W. & Kivshar Y. S. Nonlinear magnetic metamaterials. Opt. Express 16, 20266–20271 (2008). 19065165 10.1364/oe.16.020266

[b49] Carbonell J., Boria V. E. & Lippens D. Nonlinear effects in split ring resonators loaded with heterostructure barrier varactors. Microw. Opt. Technol. Lett. 50, 474–479 (2008).

[b50] Feise M. W., Shadrivov I. V. & Kivshar Y. S. Bistable diode action in left-handed periodic structures. Phys. Rev. E 71, 037602 (2005). 10.1103/PhysRevE.71.037602 15903645

[b51] Ye Y. & He S. 90° polarization rotator using a bilayered chiral metamaterial with giant optical activity. Appl. Phys. Lett. 96, 203501 (2010).

[b52] Shi Y., Yu Z. & Fan S. Limitations of nonlinear optical isolators due to dynamic reciprocity. Nat. Photonics 9, 388–392 (2015).

