# Supplementary material for: All-passive nonreciprocal metastructure
Source: Nat Commun. 2015 Sep 28;6:8359. doi: 10.1038/ncomms9359 (PMC4598563; doi:10.1038/ncomms9359)
Supplement: Supplementary Information — Supplementary Figures 1-7, Supplementary Notes 1-4 and Supplementary References. [file ncomms9359-s1.pdf]

Supplementary Information

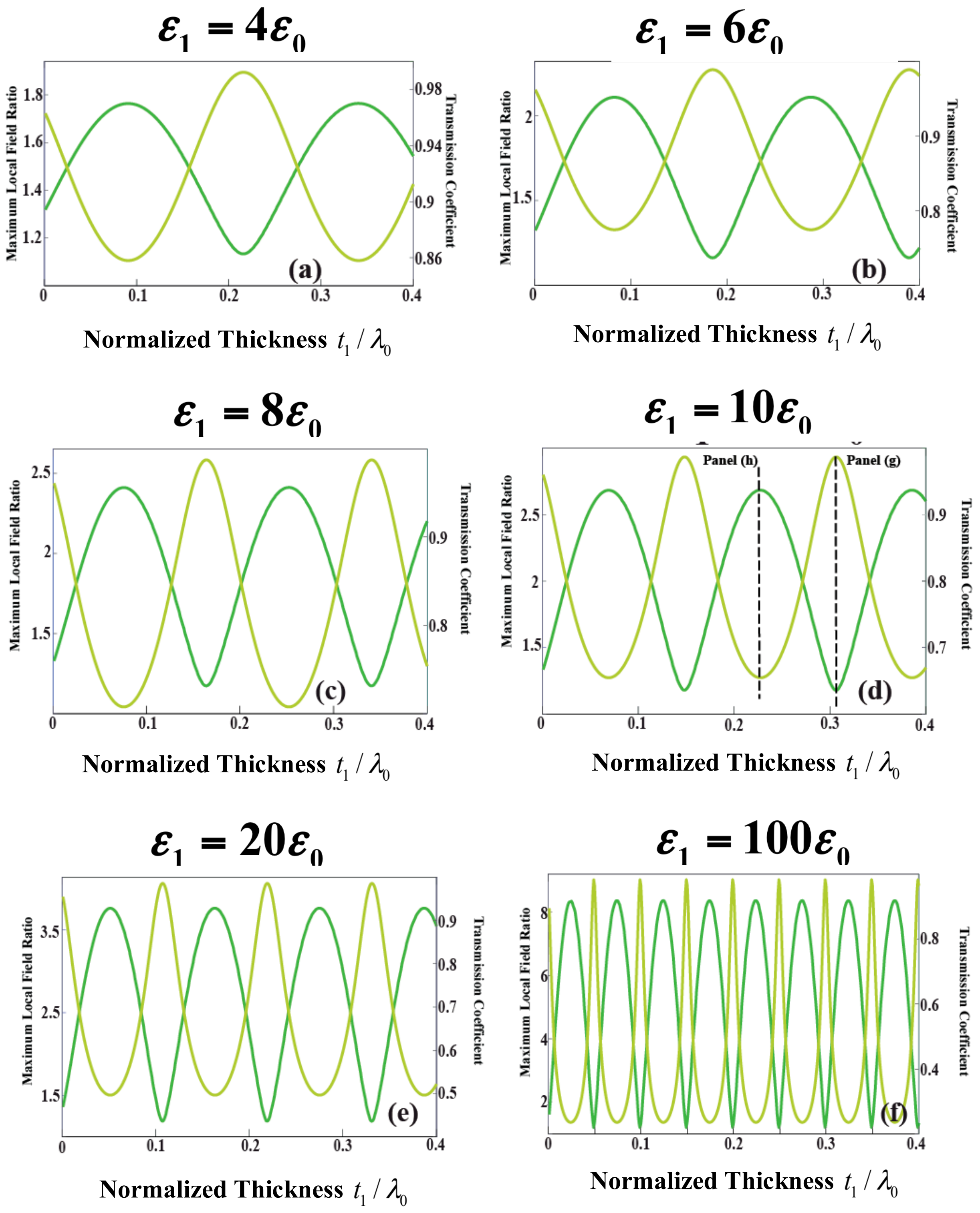

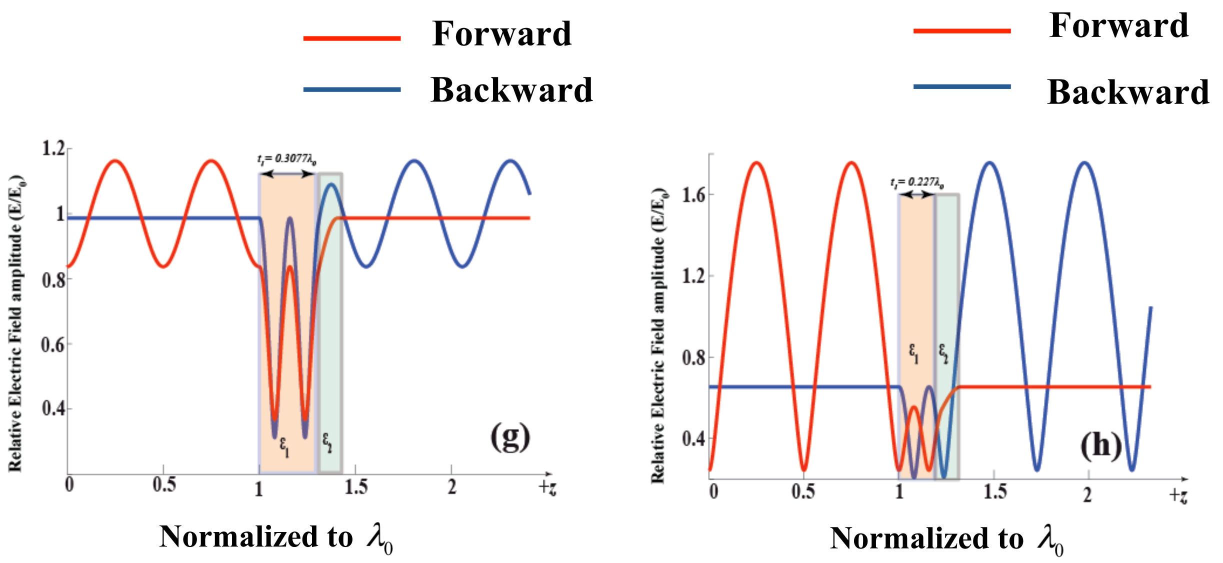

Supplementary Figure 1 ***Maximum local field ratio and transmission coefficient****. Maximum local field ratio (green curve) and transmission coefficient (yellow curve) of a pair of 1D slabs infinitely extent in the transverse directions, versus normalized thickness* t_1 *for* $t_2 = 0.1\lambda_0$, $\varepsilon_2 = 2\varepsilon_0$, *and* $\varepsilon_1 = 4\varepsilon_0$ *(a),* $6\varepsilon_0$ *(b),* $8\varepsilon_0$ *(c),* $10\varepsilon_0$ *(d),* $20\varepsilon_0$ *(e), and* $100\varepsilon_0$ *(f). The relative electric field amplitude distribution within a bilayered asymmetric one-dimensional (1D) slab infinitely extent in the transverse directions (normalized to incident field amplitude* E_0*) for: a case of close to full transmission (about 0.98 transmission coefficient) and that leads to a negligible MLFR (g), a case of 0.65 transmission coefficient and a pronounced MLFR (h).*

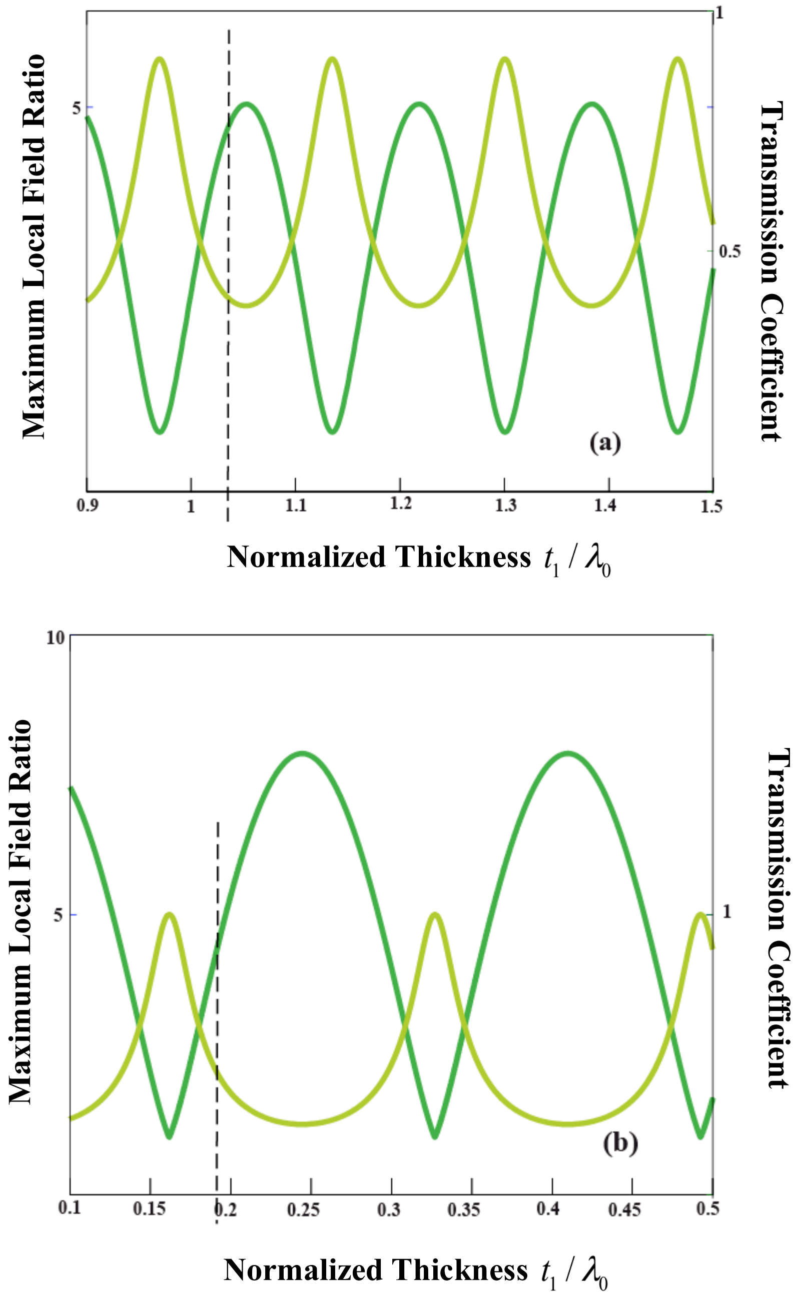

Supplementary Figure 2 **Bilayered slab inside a waveguide.** *(a) Maximum local field ratio (MLFR) (green curve) and transmission coefficient (yellow curve) versus normalized thickness* t_1 / λ_0 *with* $t_2 = 2.03\lambda_0$, $\varepsilon_1 = (10 + 0.0007i)\varepsilon_0$, *and* $\varepsilon_2 = (2 + 0.0002i)\varepsilon_0$, *based on commercially available materials, (b) Similar to (a) with* $t_2 = 0.5\lambda_0$. *The dashed lines show the operating point for which a transmission coefficient is 0.425 and MLFR is 4.4 as in the manuscript.*

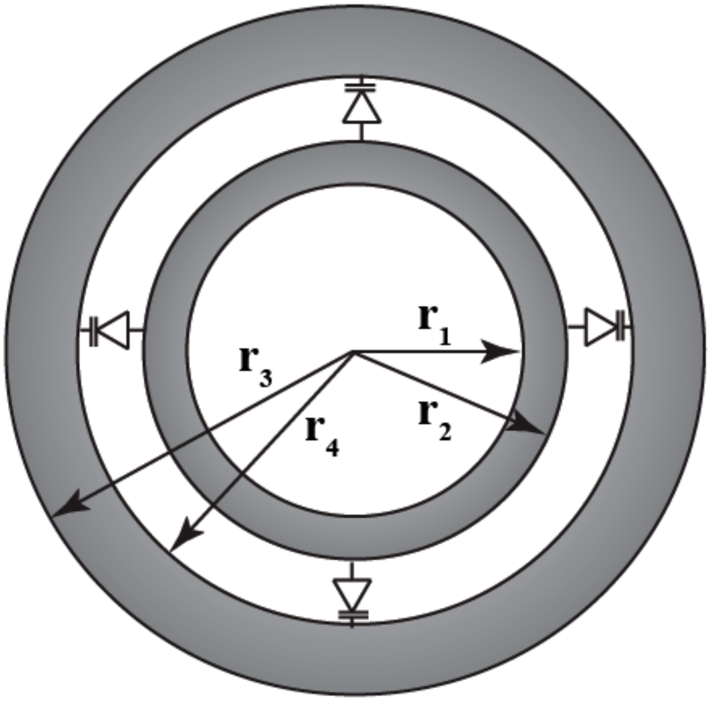

Supplementary Figure 3 ***Schematic of the nonlinear resonant structure****. It is formed of two concentric rings and loaded with four nonlinear varactors*

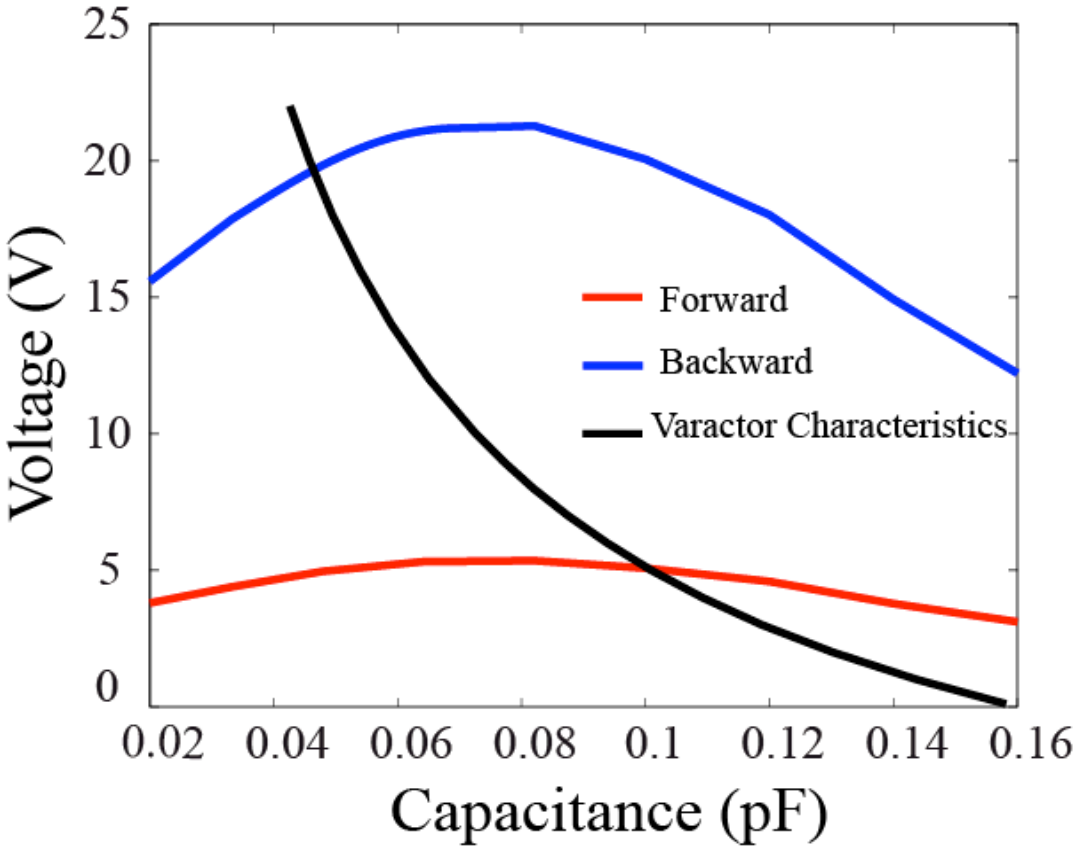

Supplementary Figure 4 ***Solution of the Nonlinear Problem for input power of 30 dBm.*** *The varactor's capacitance is swept over in the numerical simulator, and the induced voltage across the varactors is observed. The red and blue curves show the dependence of the voltage on the capacitance value of the varactors for the forward and the backward propagation, respectively. The black curve shows the C-V charactistics of the varactors [Ref. 1]. The points of intersection of the black curve with the red and the blue curves give the value of the capacitance which the varactor would exhibit in forward and backward directions, respectively*

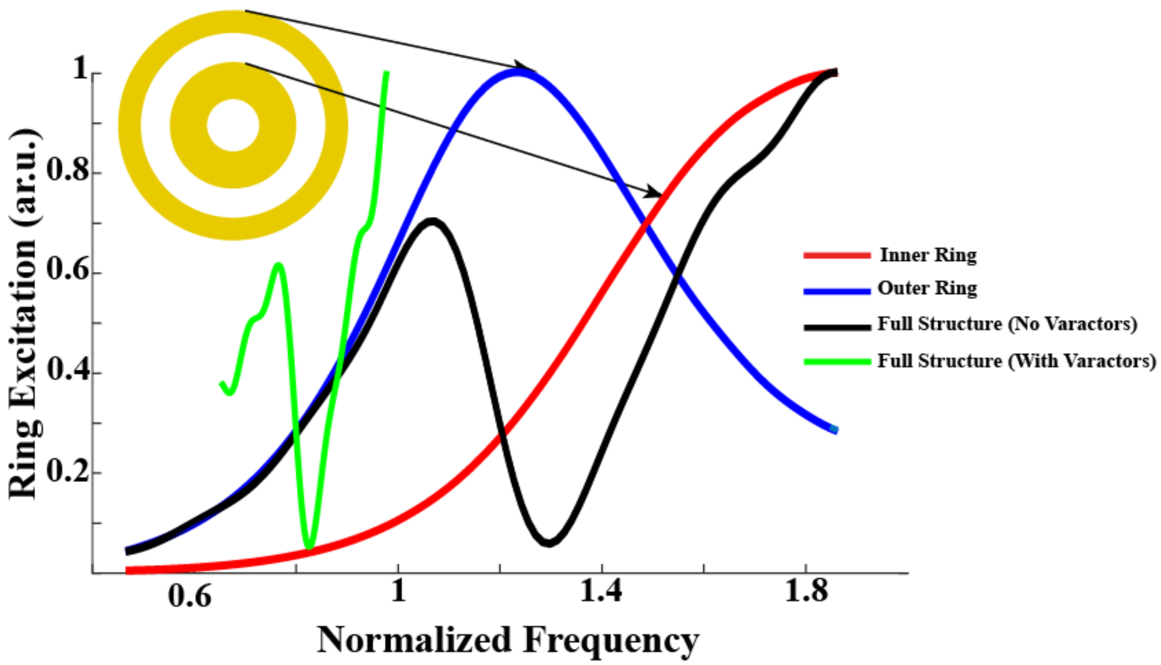

Supplementary Figure 5 ***Analysis of ring resonator resonances****. Ring excitation of inner (red curve), outer (blue curve), full structure without varactors (black curve), and full structure with varactors (green curve).*

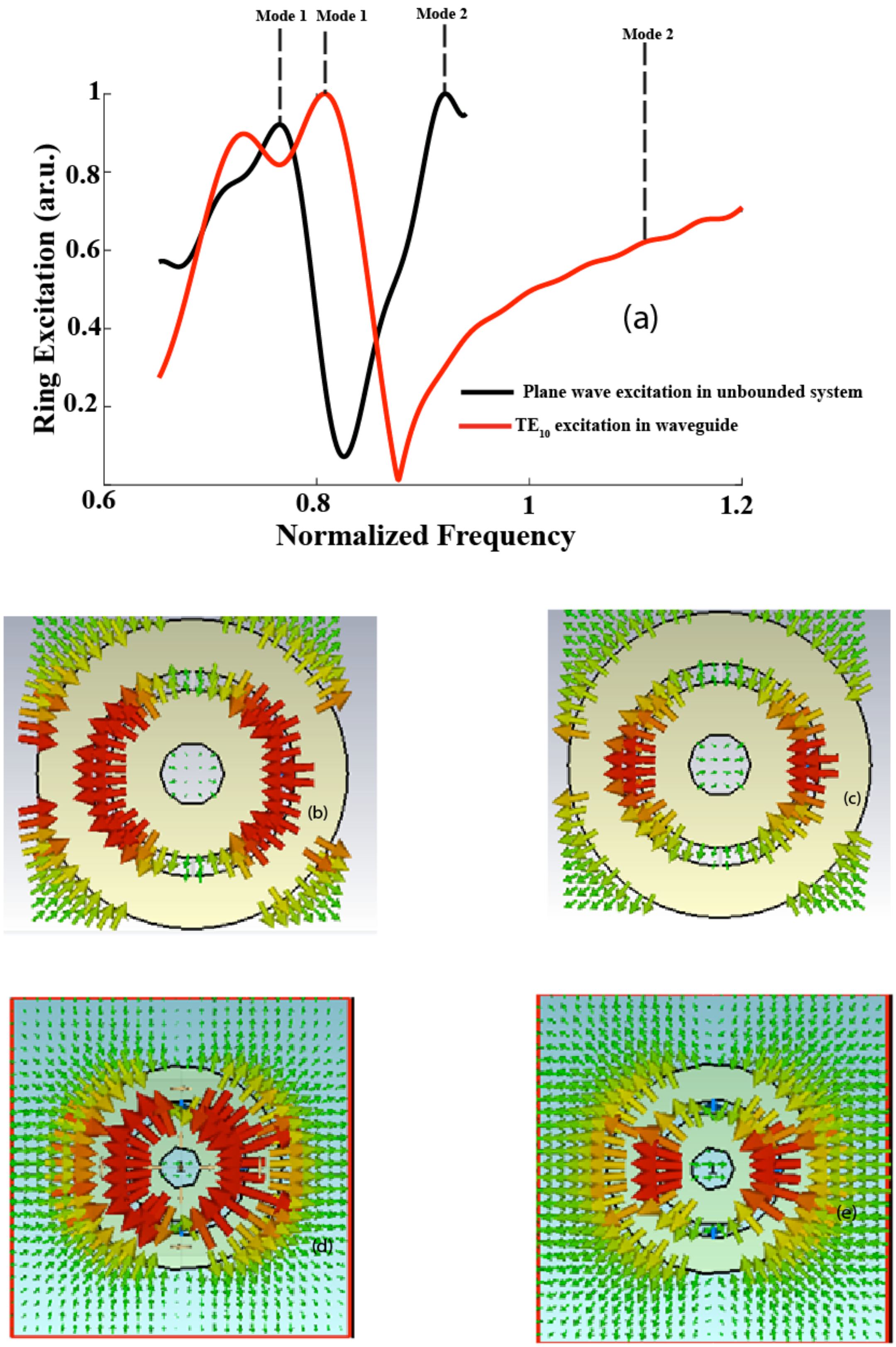
Mode 1
Mode 1
Mode 2
Mode 2
Ring Excitation (ar.u.)
Normalized Frequency
0.6
0.8
1
1.2
0.2
0.4
0.6
0.8
1
(a)
Plane wave excitation in unbounded system
TE10 excitation in waveguide
(b)
(c)
(d)
(e)

Supplementary Figure 6 ***Analysis of ring resonator resonances and eigenmodes****. (a) Ring excitation in unbounded system excited by plane wave (black curve), and inside a waveguide excited by* TE_{10}*mode (red curve), Electric field distribution across the ring in unbounded system at (b) first resonance (labeled mode 1 on black curve), (c) second resonance (labeled mode 2 on black curve), Electric field distribution across the ring in a waveguide at (d) first resonance (labeled mode 1 on black curve), (e) second resonance (labeled mode 2 on black curve)*

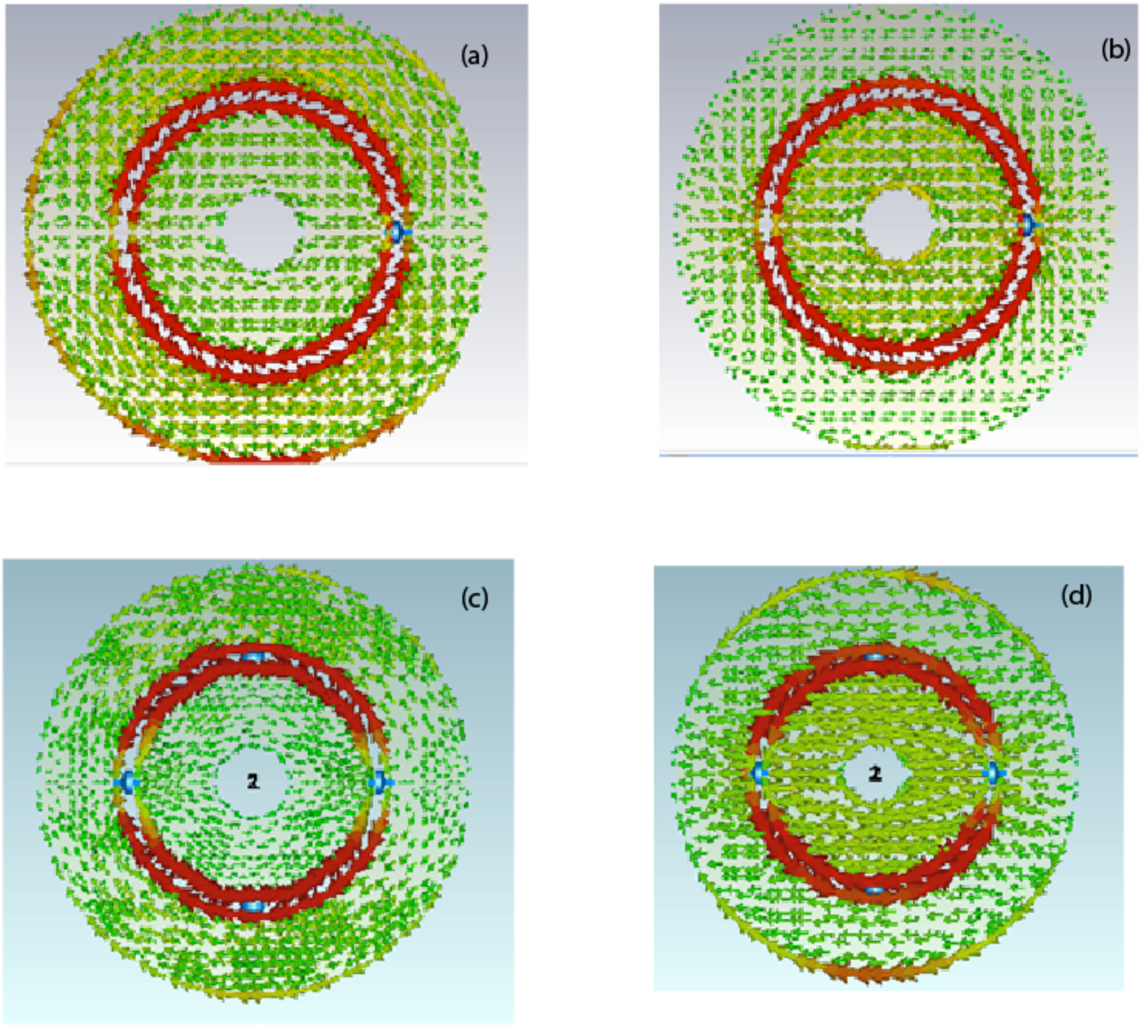

Supplementary Figure 7 ***Surface current distributions across the ring resonator****. Surface current distribution across the ring in unbounded system at (a) first resonance (labeled mode 1*

on black curve), (b) second resonance (labeled mode 2 on black curve), Surface current distribution across the ring in a waveguide at (c) first resonance (labeled mode 1 on black curve), (d) second resonance (labeled mode 2 on black curve)

Supplementary Note 1:

We start our analysis with the geometry shown in Fig. 2(a) of the manuscript. To understand the dynamics and trade-offs of such system, we consider plane waves normally incident in forward (+z) and backward (-z) directions upon the slab interface. Using transfer matrix approach we find the transmission coefficient for such a bilayered asymmetric 1D slab in addition to the field distribution within each of two slabs.

As shown in Supplementary Figure 1 we perform a study, in which we fix ε_2 to be $2\varepsilon_0$ and t_2 to be $0.1\lambda_0$, where λ_0 is the free space wavelength. Then for different values of ε_1, we sweep over t_1 and plot both the transmission coefficient and the MLFR for each case. As shown in Supplementary Figure 1, there is a trade-off between achieving high transmission coefficient and high MLFR. Moreover, the lower bound of the transmission within the system is set by the level of asymmetry between the two slabs constituting the bi-layer slab, i.e. how different are ε_1 and ε_2. For example, comparing Supplementary Figure 1(a) where $\varepsilon_1 = 4\varepsilon_0$and Supplementary Figure 1(f), where $\varepsilon_1 = 100\varepsilon_0$, ($\varepsilon_2 = 2\varepsilon_0$ for all cases as mentioned above), we can see clearly that for the former case the lower bound of the transmission coefficient is about 0.86, with a corresponding MLFR of around 1.75. However in the latter, the lower bound of the transmission coefficient is as low as around 0.25 with a corresponding MLFR of around 8.3.

As a proof of concept we choose to utilize the case depicted in Supplementary Figure 1(d) in which we can achieve both good MLFR and acceptably high transmission coefficient to further demonstrate our concept. As depicted in Supplementary Figure 1(g) if we choose to operate at the near full transmission point (around 0.98 transmission coefficient), the field distributions inside the structure illuminated from both sides are almost identical and we do not achieve any significant MLFR. However if we choose to work at the lower bound of transmission (that is around 0.65 coefficient), this corresponds to an MLFR of around 2.733, which is clearly depicted in Supplementary Figure 1(h). Note that throughout the manuscript we utilize a bilayered slab of larger thickness (around $3\lambda_0$, and both as 1D slabs (Figs. 2(a) and 2(b) and inside the waveguide (Figs. 2(c) and 3(a)) than what is shown in this section mainly for the sake of visual clarity throughout all the figures. As shown here, however, we can achieve somewhat similar transmission coefficient and MLFR reported in the manuscript (which directly translates into an acceptably low insertion loss accompanied by a good nonreciprocal transmission ratio) using a structure of a total thickness of around $0.327\lambda_0$ (for the 1D slab case) with the same set of dielectric constants. This verifies that in principle our approach may apply for subwavelength structures.

Supplementary Note 2:

In Supplementary Figure 2(a) we repeat Fig. 2(b) of the main manuscript but this time within the waveguide defining MLFR and transmission coefficient for the $0.54\lambda_0 \times 0.54\lambda_0$ waveguide and using realistic commercially available materials with their losses included. In Supplementary Figure 2(a) we have, $\varepsilon_1 = (10 + 0.0007i)\varepsilon_0$ which is commercially available from Emerson & Cuming under the set of materials labelled “ECCOSTOCK HiK500F” , $\varepsilon_2 = (2 + 0.0002i)\varepsilon_0$ which is the commercially available Teflon AF 1600 that can be purchased

from (among many other companies) *Dupont* and $t_2 = 2.03\lambda_0$. We plot the MLFR along with the corresponding transmission coefficient versus the variation of the first layer t_1 / λ_0. The analysis shows that when $t_1 = 1.03\lambda_0$ we achieve a transmission coefficient of 0.425 and an MLFR of 4.4, which are the values utilized in our nonlinear diode in a rectangular waveguide example. We note that similar analysis is shown in Supplementary Figure 2(b), however limiting the thickness of the bilayered slab to be subwavelength by choosing $t_2 = 0.5\lambda_0$ and varying t_1 / λ_0. A similar transmission coefficient and MLFR to the ones used in the manuscript can be achieved for $t_2 = 0.19\lambda_0$ as shown.

Supplementary Note 3:

The nonlinear resonant layer is formed of two concentric rings with r_1, r_2, r_3, and r_4 being $0.03\lambda_0$, $0.08\lambda_0$, $0.0975\lambda_0$, and $0.1475\lambda_0$respectively as shown in Supplementary Figure 3. The rings are made of perfect electric conductor (PEC) and have a thickness of $0.0004\lambda_0$ for the purpose of our numerical simulation. The resonator is loaded with four nonlinear varactors as shown in Fig. 2 in the manuscript and Supplementary Figure 3. Such a unit cell is suitable for a diode-like operation that is polarization-angle independent, as well as having a small footprint for a specific resonance frequency, as compared with a single ring resonator. The resonator is then incorporated at the MLFR location, such that when the resonator is fed from one side depending on the power level its resonance frequency may be different from the resonance frequency when fed from the other side, owing to the change of the varactor's capacitance. This is due to the fact that the local fields caused by excitations from the two sides are different. The dimensions of the resonator itself are chosen such that it is almost "transparent" (i.e., low scattering at off-resonance) when the varactor is operated in the linear regime being excited with low power, and as the power level is increased, it moves towards being "opaque" (i.e., high

scattering at resonance). Therefore, when excited from the side that induces relatively low local field at the varactor's location, the resonator is still "transparent" and we get a relatively high transmission, which is about 0.42 in this design for an input power of 30 dBm. On the other hand, when the structure is excited from the other side using the same power level, that induces high local field at the varactor's location, the resonator turns to be "opaque" and the wave transmission in that direction is much less.

The varactor diode used in our design is the so-called Heterostructure Barrier Varactor (HBV) Ref [1], which exhibits a symmetric C (V) characteristic. It is clear that such symmetry property allows use of an unbiased device and the capacitance control utilizing a moderate signal. The plots shown in Fig. 3(b) and (c) in the manuscript are obtained using Time Domain Solver of CST Microwave Studio®, when the ring is inside the waveguide filled with only the material with permittivity $\varepsilon_2 = 2\varepsilon_0$, and the value of capacitance of varactor is selected based on what it should be when the loaded ring is inserted in the bilayered dielectric slab and illuminated in the forward direction (when the capacitance is $C_f = 0.157pF$ for incident power level 5 dBm, and $C_f = 0.1pF$ for incident power level 30 dBm) and in the backward direction (when the capacitance is $C_b = 0.147pF$ for incident power level 5 dBm, and $C_b = 0.05pF$ for the power level 30 dBm). Figures 3(b) and 3(c) show the resonance behavior of the ring when loaded with these various capacitance values. Fig. 3(d) is obtained when the ring is inserted in the waveguide with the bilayered dielectric slab present. The utilized geometry is as shown in Fig. 2(c) of the manuscript where we have a metallic waveguide with a square cross section $0.54\lambda_0$ x $0.54\lambda_0$, where λ_0 is the free space wavelength, that is loaded with two dielectric slabs with relative permittivities $\varepsilon_1 = 10\varepsilon_0$ and $\varepsilon_2 = 2\varepsilon_0$, and thicknesses, $t_1 = 1.03\lambda_0$ and $t_2 = 2.03\lambda_0$. A waveguide

port is used to excite the system with the TE_{10} mode of the waveguide using a power ranging from 3.126 mW (5 dBm) to 1 W (30 dBm). For each power level, the dynamic C (V) characteristics of the HBV is then plotted versus the induced voltage across the varactors exciting it from both ends of the waveguide, while sweeping over different values of capacitances. The operating point can then be found where the curves intersect (see Supplementary Figure 4, which is the plot for the 30 dBm power level). The values are found to be around $C_b = 0.05pF$ and $C_f = 0.1pF$ for a power of 1W (30 dBm), where C_b and C_f are the capacitance values of the varactor for the cases of backward and forward incident waves, respectively. These correspond to a negligible transmission coefficient for backward travelling wave and 0.42 for a forward travelling wave. The same approach is used to find the transmission coefficients in forward and backward directions at every power level as shown in Fig. 3(d) of the manuscript where the ring is in the waveguide with the dielectric slabs present.

Supplementary Note 4:

The shape of the resonance curve is due to the interaction between two ring eigenmodes with different symmetries. Note that an analogous system has been studied and classified using hybridization in [2] , where the modes were classified based on the induced charge distribution and the shape of the resonance curve was explained based on a hybridization model. In Supplementary Figure 5 we show a similar analysis for our structure, which analogously resembles the results in [2].

We further note that our analysis shows that qualitatively the ring response is the same inside the waveguide and for the case of a plane wave illumination, see Supplementary Figure 6(a). In both cases the excitation of two modes (symmetric and antisymmetric) is clearly seen. The

corresponding field distribution is shown in Supplementary Figures 6(b), and (c) for the plane wave illumination and in Supplementary Figures 6(d) and (e) for the waveguide excitation. We stress that the mode configuration is determined by the induced charge distribution (i.e., electric field map) and not the distribution of the surface current in the rings. Furthermore, our analysis of the surface current (see Supplementary Figure 7) shows that the surface current distribution is always antisymmetric. And indeed, only anti-parallel current distribution excites the varactors. But we note that this configuration is always excited in our system, even in the case of a plane wave (where there is no longitudinal magnetic field). Thus our design is not limited only to TE modes and can be used for infinitely extended structures excited with plane waves.

Supplementary References

1. Carbonell, J., Boria, V. E. & Lippens, D. Nonlinear effects in split ring resonators loaded with heterostructure barrier varactors, *Microw. Opt. Technol. Lett.* **50**, 474–479 (2008).
2. Prodan, E., Radloff, C., Halas, N. J. and Nordlander, P. A hybridization model for the plasmon response of complex nanostructures, *Science*, **302**, 419–422 (2003).
